# Maternal sex influences seed dormancy in a gynodioecious species

**DOI:** 10.1093/aobpla/plag031

**Published:** 2026-07-03

**Authors:** Brooklyn A Richards, F Andrew Jones, Thomas N Kaye

**Affiliations:** Department of Botany and Plant Pathology, Oregon State University, 2701 SW Campus Way, 2503 Cordley Hall, Corvallis, OR 97331, United States; Department of Botany and Plant Pathology, Oregon State University, 2701 SW Campus Way, 2503 Cordley Hall, Corvallis, OR 97331, United States; Smithsonian Tropical Research Institute, Apartado Postal 0843-03092, Panamá, República de Panamá; Department of Botany and Plant Pathology, Oregon State University, 2701 SW Campus Way, 2503 Cordley Hall, Corvallis, OR 97331, United States; Institute for Applied Ecology, 4950 SW Hout St, Corvallis, OR 97333, United States

**Keywords:** dormancy, female advantage, germination, gynodioecy, seed quality, *Sidalcea campestris*

## Abstract

Maternal genotype and environment can influence seed characteristics, yet sex-specific maternal effects in gynodioecious systems remain poorly understood. We evaluated how maternal sex affects seed viability, mass, and dormancy in the gynodioecious plant, *Sidalcea campestris*. We compared seed viability and mass to determine whether differences in seed quality contribute to germination variability and female advantage. Seeds were scarified by manually nicking the seed coat with a razor blade and cold stratified at 5°C. Germination of seeds with and without scarification across 0, 6, and 12 weeks of cold stratification was evaluated in order to test how maternal sex affects dormancy. Seed viability and mass did not differ between sexes, suggesting that these traits do not contribute to female advantage in this system. Scarification and cold stratification increased germination, indicating germination may be influenced by physical and physiological dormancy. However, the effect of dormancy-breaking treatments on germination depended on maternal plant sex, with seeds from hermaphrodites being more easily released from dormancy than seeds from females. Seeds from hermaphrodites germinated significantly more than seeds from females when scarified, with probabilities 119% higher at 0 weeks and 18% higher at 12 weeks of stratification. Such differences may have consequences on the timing of germination and survival of seeds produced by females and hermaphrodites, with effects that may vary across time and space. Further work is needed to understand how sex-specific maternal influence on dormancy shape the ecology and evolution of gynodioecy.

## Teaser

We tested whether female and hermaphrodite *Sidalcea campestris* plants produce seeds that differ in quality or germination behaviour. Seed viability and mass were similar between sexes, suggesting no differences in basic seed quality. However, treatments that break dormancy affected sexes differently: hermaphrodite seeds germinated more readily than female seeds after scarification and cold exposure. These differences may influence when seeds germinate and how offspring establish in natural populations.

## Introduction

The evolution of dimorphic plant sexual systems has intrigued ecologists and evolutionary biologists since Darwin’s time (1877). Today, it remains relevant to research on agriculture (Delph et al. 2007, Chen and Liu 2014), environmental change (Hultine et al. 2016, Varga and Soulsbury 2020, Hangartner et al. 2022), ecological interactions (Ågren et al. 1999, Ashman 2006), species diversification (Käfer et al. 2014, Sabath et al. 2016), and plant trait evolution more broadly (Eckhart 1999, Ashman et al. 2013, Renner 2014, Barbot et al. 2023).

The coexistence of females and hermaphrodites, or gynodioecy, is a plant sexual system that may function either as an intermediate stage in the evolution from hermaphroditism to dioecy (Spigler and Ashman 2012, Dufay et al. 2014) or as an evolutionarily stable state (Charlesworth and Charlesworth 1978, Dufaÿ et al. 2007, Pannell and Jordan 2022). Females in gynodioecious populations must compensate for the fitness cost of only contributing genetically via ovules, by having a reproductive advantage in either offspring quantity or quality. The level of reproductive advantage required for females to persist depends on the mode of sex determination, ranging from a twofold advantage under purely nuclear inheritance (Charlesworth and Charlesworth 1978) to only a modest advantage (>1) under the more common cytonuclear systems, where sex is determined by interactions between cytoplasmic male sterility (CMS) genes and nuclear restorer (*Rf*) alleles (Bailey et al. 2003, Bailey and Delph 2007a). Although empirical evidence supports female advantage in a variety of gynodioecious systems, the majority of this research has emphasized quantitative differences in offspring (Shykoff et al. 2003, Dufay and Billard 2012). In contrast, the importance of qualitative differences in offspring to maintaining female advantage remains poorly understood (Varga 2021).

Variation in seed fitness influences survival and recruitment (Baskin and Baskin 2014), which could have important effects on the population dynamics of sexually dimorphic plants (Purrington and Schmitt 1995). Support for differences in seed quality such as mass, germination, and nutritional content is reported in some gynodioecious species (Shykoff et al. 2003, Varga 2021) but not in others (Ashman 1994, Delph and Mutikainen 2003, Baskin and Baskin 2020, Varga 2021). Even if females in gynodioecious populations maintain a reproductive advantage in terms of seed quantity (i.e. producing more seeds), qualitative fitness components have the potential to enhance or counteract such advantage.

Maternal genotype and environment can have a strong influence on seed characteristics (Baskin and Baskin 2014). In this study, we evaluated the effect of maternal sex on seed dormancy in *Sidalcea campestris*. While previous studies have compared germination between maternal sexes in gynodioecious species (Baskin and Baskin 2020), including *Sidalcea* (Ashman 1992, Marshall and Ganders 2001), our study is the first to our knowledge to experimentally test whether seeds produced by female and hermaphrodite plants differ in their dormancy properties. Specifically, we used cold stratification and scarification treatments to answer the following research questions. First, we tested whether seed quality, assessed using seed mass and viability, differs between the sexes to evaluate whether variation in seed dormancy could be attributed to differences in seed quality. Second, we evaluated germination responses to scarification and cold stratification to infer potential dormancy mechanisms in this species. Finally, we tested whether dormancy differs between seeds produced by female and hermaphrodite plants.

## Materials and methods

### Study species

**Figure 1:**
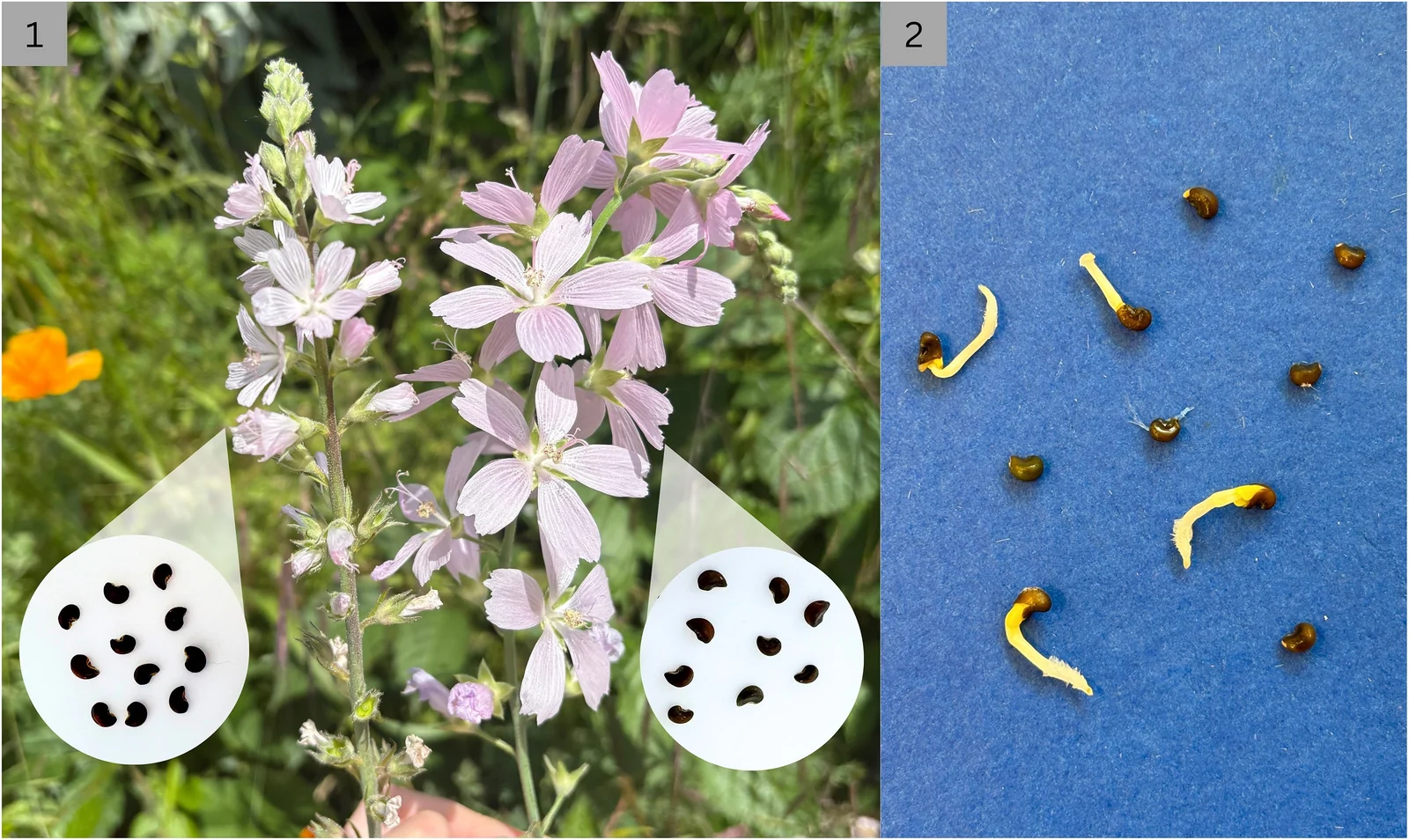
Study species and germination setup. (1) Flowers and seeds of female (left) and hermaphrodite (right) *Sidalcea campestris* plants. (2) Germination box containing 10 seeds per replicate, as used in dormancy experiments.

*Sidalcea campestris* Greene is a hexaploid, insect-pollinated mallow species endemic to Oregon’s Willamette Valley. Like most *Sidalcea* species, *S. campestris* has a gynodioecious sexual system, with individuals producing either perfect or pistillate flowers, referred to as hermaphrodites and females, respectively (Fig. 1, Box 1). Hermaphrodites produce larger flowers with both pollen and nectar rewards that are preferentially visited by pollinating insects (Richards et al. 2026a). Female flowers are smaller, produce only nectar, and are obligately outcrossed. At maturity, the dry schizocarp fruit breaks apart into 6–9 single-seeded mericarps. Seed dormancy is often reported in *Sidalcea* (Halse and Mishaga 1988, Russell 2011, Jones and Kaye 2015). Seed production in both sexes is pollen limited, and females achieve a higher seed set (Richards et al. 2026b) and experience reduced pre-dispersal seed predation (Richards et al. 2026a). The wide variation in population female frequency (7%–75%) suggests that sex determination in *S. campestris* likely follows cytonuclear inheritance (Bailey and Delph 2007a), consistent with findings in other *Sidalcea* species (Ashman 1992, Graff 1999).

### Seed quality

Seeds for this study were harvested June–July, 2024 from female and hermaphrodite plants in three natural *S. campestris* populations located in western Oregon’s Willamette Valley (Supplementary Table S1). To estimate seed viability, seeds were assessed with a tetrazolium (TZ) test at the Oregon State University Seed Lab following standard procedures outlined by the Association of Official Seed Analysts (AOSA). The TZ test is a biochemical assay that determines seed viability based on dehydrogenase activity. This test allowed us to compare germination levels in our dormancy-breaking treatments to baseline seed viability. For each maternal sex, 225 seeds were pooled in equal numbers from the three source populations. Of these, 208 female seeds and 207 hermaphrodite seeds were scored for viability using standard procedures (Kearns and Inouye 1993). We compared seed viability between maternal sexes using a chi-squared test.

We measured seed mass by weighing 30 seeds per site for each maternal sex to assess whether mass differences could contribute to observed germination patterns. Individual seeds were weighed using an analytical balance (OHAUS Analytical Plus AP110, Ohaus Corporation, Florham Park, New Jersey, USA). We analysed differences in seed mass between maternal sexes using a generalized linear mixed-effects model (GLMM) with a gamma distribution (glmmTMB package in R; Brooks et al. 2017). The response variable was individual seed weight, and maternal plant sex was included as a fixed effect. Origin site was included as a random effect to account for non-independence of seeds collected from the same site.

### Dormancy treatments

Prior to treatment, all seeds were stored in dry conditions for ∼6–7 months at room temperature. While after-ripening during storage may influence dormancy status, all seed lots experienced the same storage conditions before experimentation. For each site and sex, seeds were randomly assigned a scarification treatment (scarified vs. not scarified) and cold stratification treatment (0, 6, and 12 weeks). Each treatment combination included four replications of 10 seeds (*n* = 1440). Sample sizes were constrained by natural variation in seed production and high levels of pre-dispersal seed predation in source populations. All seeds were placed in germination boxes on wet blotter paper (Fig. 1, Box 2). Seeds were scarified by manually nicking the seed coat with a razor blade. Cold stratified seeds were held at 5°C for their respective treatment time. Seeds were transferred to a growth chamber after treatments were applied, where they experienced a daily cycle of 12 h of light at 25°C and 12 h of darkness at 15°C. Blotter paper was misted as needed to maintain germination box moisture. Seeds with an emerged radicle >1 mm were considered germinated. Germination was assessed weekly over a 2-week period (Gisler 2003, Jones and Kaye 2015). In addition to final germination proportion, we quantified germination dynamics over time using weekly counts and constructed cumulative germination curves. Germination timing was summarized using T50 (time to 50% germination), calculated for each germination box and summarized by treatment.

To test for the effect of maternal plant sex on dormancy-breaking requirements, we used a GLMM with a binomial distribution (lme4 package in R; Bates et al. 2015). The response variable was the proportion of germinated seeds. We modelled plant sex, scarification, and stratification treatments as fixed effects, including interactions between maternal plant sex and each dormancy-breaking treatment. Origin site and germination box were included as random effects. We performed post-hoc pairwise comparisons using estimated marginal means (emmeans package in R; Lenth and Piaskowski 2026) to compare germination between females and hermaphrodites across treatment combinations.

## Results

**Figure 2:**
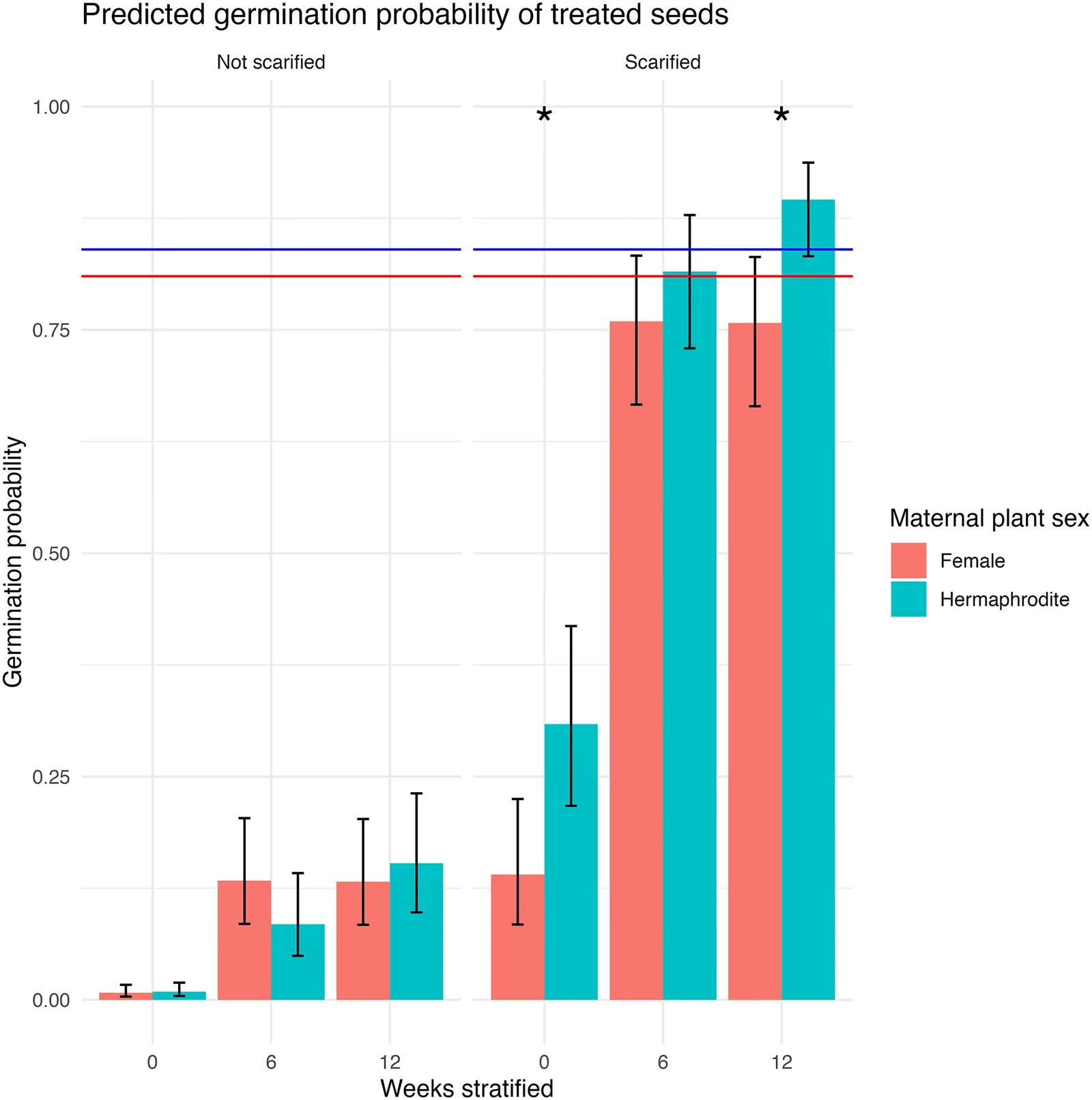
The predicted germination probability of sidalcea campestris seeds from female (red) and hermaphrodite (blue) plants with and without scarification across 0, 6, and 12 weeks of cold stratification. The error bars represent 95% confidence intervals, and the black asterisks indicate a significant difference between sexes (*P* ≤ 0.05). Predicted probabilities are based on post-hoc pairwise comparisons of estimated marginal means from the GLMM. The horizontal lines indicate seed viability for females (red) and hermaphrodites (blue) assessed using a TZ test.

Seed viability, assessed using a TZ test, did not differ significantly between maternal sexes (females 81%; hermaphrodites 84%; *χ*^2^ = 0.77, *P* = 0.45). Predicted germination probabilities for scarified seeds subjected to 6 or 12 weeks of cold stratification (females: 0.759 and 0.758; hermaphrodites: 0.815 and 0.896, respectively) were close to the baseline viability estimates from the TZ test, which fell within the 95% confidence intervals of the model predictions (Fig. 2). There was no evidence that seed mass differed between the two sexes (*z* = 0.16, *P* = 0.875), suggesting that the differences in dormancy and germination observed between sexes were not explained by seed mass.

**Figure 3:**
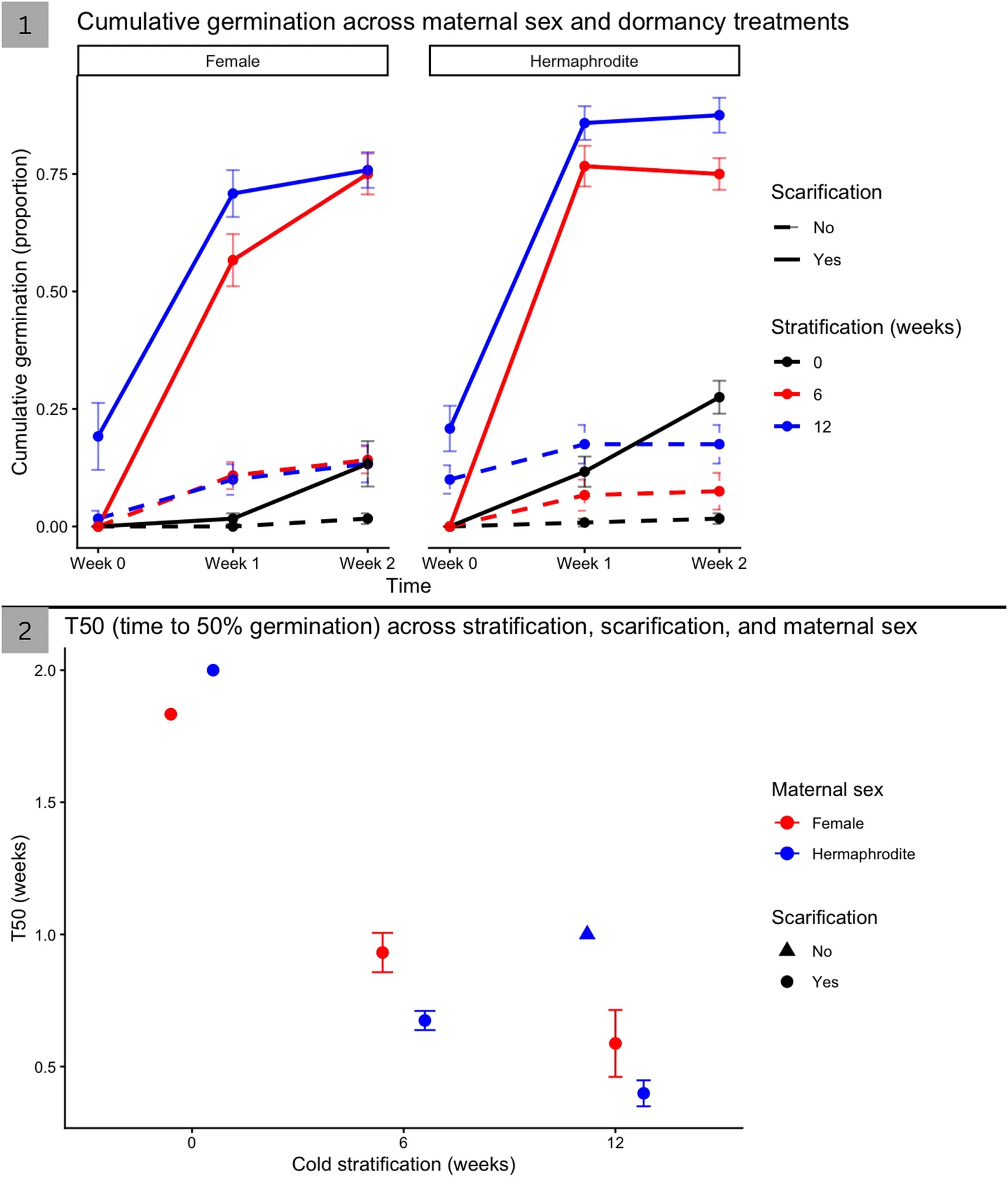
Germination dynamics and germination timing across maternal sex and dormancy-breaking treatments in *Sidalcea campestris*. (1) Cumulative germination curves across time (0, 1, and 2 weeks after transfer to growth chamber; week 0 represents germination recorded at the time of transfer to the growth chamber) for seeds from female and hermaphrodite maternal plants subjected to combinations of scarification (scarified vs. not scarified) and cold stratification (0, 6, and 12 weeks). Values represent mean proportion germination per germination box ± SE. (2) Germination timing expressed as T50 (time to 50% germination) across the same treatment combinations. T50 was calculated at the level of individual germination boxes and summarized by treatment. In several treatment combinations, germination did not reach 50% within the experimental period, resulting in no estimable T50 values (*n* = 0), which are not shown. In cases where only a single germination box reached 50% germination (*n* = 1), T50 is displayed but associated variability (SE) is not presented due to insufficient replication for variance estimation.

For both females and hermaphrodites, the highest germination levels were observed in the scarified + 12-week cold stratification treatment group, with an average germination proportion of 0.758 and 0.875, respectively (Table 1). The average germination proportion for females was similar in the stratified + 6-week cold stratification treatment group, at 0.75. Most germination occurred within the first week after transfer to the growth chamber (Fig. 3, Box 1). T50 was strongly influenced by dormancy-breaking treatments (Fig. 3, Box 2). T50 could not be estimated for most non-scarified treatments, as germination did not reach 50% within the monitoring period. The only exception was a single germination box from the 12-week stratified, non-scarified hermaphrodite treatment. Among scarified treatments, T50 decreased with increasing cold stratification duration, indicating faster attainment of 50% germination following longer stratification. In females, mean T50 (±SE) declined from 0.932 ± 0.07 weeks under 6-week stratification to 0.588 ± 0.13 weeks under 12-week stratification. A similar pattern was observed in hermaphrodites, with T50 decreasing from 0.674 ± 0.04 weeks (6 weeks stratification) to 0.399 ± 0.05 weeks (12 weeks stratification).

**Table 1:** Germination responses of *Sidalcea campestris* seeds across maternal sex, scarification, and cold stratification treatments.

Maternal sex	Scarified	Weeks stratified	Total germinated (count)	Mean germination per box (±SD)
Female	No	0	2	0.17 (±0.39)
Female	No	6	17	1.42 (±1.00)
Female	No	12	16	1.33 (±1.37)
Female	Yes	0	17	1.42 (±1.68)
Female	Yes	6	90	7.5 (±1.51)
Female	Yes	12	91	7.6 (±1.31)
Hermaphrodite	No	0	2	0.17 (±0.39)
Hermaphrodite	No	6	9	0.75 (±1.37)
Hermaphrodite	No	12	21	1.75 (±1.42)
Hermaphrodite	Yes	0	37	3.08 (±1.51)
Hermaphrodite	Yes	6	99	8.25 (±1.48)
Hermaphrodite	Yes	12	105	8.75 (±1.29)

Total germinated (count) represents the number of seeds germinated out of 120 seeds per treatment combination. Mean germination per box (±SD) represents the mean number of germinated seeds per germination box (out of 10 seeds), calculated across 12 replicate boxes per treatment combination.

Our model shows that cold stratification (both 6 and 12 week, relative to 0 week) and scarification each increased germination probability (*P* < 0.0001). The effect of dormancy-breaking treatments on germination depended on maternal plant sex. Model results indicated a significant interaction between maternal plant sex and scarification (*P* = 0.035).

Predicted germination probabilities, estimated using emmeans, indicated that no differences were detected between sexes among non-scarified seeds; however, scarified seeds from hermaphrodites germinated significantly more than scarified seeds from females at 0 and 12 weeks of cold stratification. Specifically, the germination probability of scarified seeds from hermaphrodites was higher than that of females by approximately 119% at 0 weeks and 18% at 12 weeks of cold stratification (Table 2; *P* = 0.007 and 0.004, respectively). Averaging across stratification treatments, seeds from hermaphrodite plants germinated approximately 33% more than seeds from female plants following scarification (*P* = 0.0005). Averaging across scarification treatments, seeds from hermaphrodite plants germinated significantly more than seeds from females at 12 weeks (*P* = 0.042), representing an approximate 36% higher germination probability.

**Table 2:** Estimated germination probabilities of seeds from females and hermaphrodites from post-hoc pairwise comparisons (emmeans package in R).

	Female	Hermaphrodite
Scarified	*0.540 (0.459–0.619)	*0.720 (0.647–0.783)
Non-scarified	0.054 (0.034–0.084)	0.051 (0.032–0.081)
Cold stratification: 0 weeks	0.035 (0.019–0.063)	0.061 (0.036–0.106)
Cold stratification: 6 weeks	0.411 (0.319–0.510)	0.390 (0.291–0.500)
Cold stratification: 12 weeks	*0.409 (0.316–0.508)	*0.555 (0.446–0.659)

	Scarified	Non-scarified	Scarified	Non-scarified
Cold stratification: 0 weeks	*0.141 (0.084–0.223)	0.008 (0.004–0.017)	*0.309 (0.218–0.419)	0.009 (0.004–0.019)
Cold stratification: 6 weeks	0.759 (0.666–0.833)	0.134 (0.085–0.203)	0.815 (0.729–0.879)	0.085 (0.049–0.142
Cold stratification: 12 weeks	*0.758 (0.665–0.832)	0.132 (0.084–0.203	*0.896 (0.832–0.937)	0.153 (0.098–0.231)

The 95% CI is included in parentheses. Estimates with one asterisk (*) indicate a statistically significant difference between sexes (*P* ≤ 0.05).

## Discussion

### Seed quality

We found that maternal sexes differed in their responses to dormancy-breaking treatments, but we detected no differences in seed viability or seed mass. Previous research on *S. campestris* supports that higher seed set (Richards et al. 2026a) and reduced pre-dispersal seed predation (Richards et al. 2026b) may contribute to female advantage. Our results suggests that qualitative differences such as seed viability and mass do not contribute to female advantage in this system. Consistent with these results, meta-analyses by Baskin and Baskin (2020) as well as Varga (2021) did not find that females in gynodioecious species necessarily produce heavier seeds. Many gynodioecious species show differences in germination between maternal sexes (Ashman 1992, Dinnetz and Jerling 1997, Shykoff et al. 2003, Varga 2021). However, given that our study is the first to test dormancy-breaking requirements between maternal sexes, we suggest that some of these differences may reflect variation in seed dormancy characteristics rather than seed quality itself.

Females are expected to experience qualitative advantages in offspring fitness due to reduced selfing and inbreeding depression (Charlesworth and Charlesworth 1978, Dornier and Dufay 2013). While avoidance of inbreeding promotes female advantage in some species, it does not appear to be a crucial component to the maintenance of gynodioecy in many cases (Ashman 1992, Dufay and Billard 2012, Dalton et al. 2013). In some *Sidalcea* species, seeds produced by females are reported to have higher germination than seeds produced by hermaphrodites regardless of inbreeding levels (Ashman 1992, Marshall and Ganders 2001). Although we did not assess inbreeding depression in our study, the observed similarity in seed viability and mass between maternal sexes suggests that any such effects, if present in natural populations of *S. campestris*, are not detectable at this early life stage.

### Seed dormancy

Overall, both scarification and stratification promoted germination, suggesting physical as well as physiological dormancy mechanisms may influence germination in *S. campestris*. However, direct tests of seed coat permeability are needed to determine dormancy class. Germination was highest in both sexes when seeds received a combination of scarification and cold stratification for 12 weeks. Among scarified seeds, longer cold stratification reduced T50, indicating that seeds not only achieved higher final germination but also germinated more rapidly following prolonged cold stratification. Our results are consistent with findings by Jones and Kaye (2015), who reported that germination was highest in *S. malviflora* ssp. *virgata* when seeds were both scarified and cold stratified for 4 weeks or more. A previous study of *S. campestris* found that seeds required cold stratification to germinate, though the author suggests that this may be due to softening of the seed coat rather than physiological dormancy, as no scarification treatments were applied (Russell 2011). Manual scarification is commonly used to promote germination in *Sidalcea* (Halse and Mishaga 1988, Ashman 1992, Marshall and Ganders 2001, Gisler 2003). Physical dormancy is common in Malvaceae, though combinational dormancy is also reported in some species (Baskin and Baskin 2014, 2025). Under natural conditions physical dormancy in Malvaceous seeds is typically overcome by dislodging of the seed’s chalazal plug via environmental fluctuations in temperature and/or moisture (Baskin and Baskin 2014, Jones and Kaye 2015). Fire may also help seeds overcome dormancy (Baskin and Baskin 2014), and there are some reports that *Sidalcea* responds positively to fire, though further research is needed (Zimmerman and Reichard 2005, Oregon Fish and Wildlife Office 2015, Fertig 2022).

### Sex-specific differences

We found sex-specific maternal effects on seed dormancy in *S. campestris*. Scarified seeds from hermaphrodites had significantly higher estimated germination probability than female seeds at both 0 and 12 weeks of stratification, suggesting that seeds from hermaphrodites were more easily released from dormancy than seeds from females. On average, hermaphrodite seeds had a 33% higher estimated germination probability than females when scarified, and a 36% higher estimated germination probability than females after 12 weeks of cold stratification. Maternal plants can affect offspring characteristics via genetic inheritance and environmental interactions (Baskin and Baskin 2014). Below, we discuss several non-mutually exclusive explanations for these sex-specific effects.

In gynodioecious plants, maternal inheritance of the mitochondrial genome facilitates the spread of CMS mutations, which gives rise to the male-sterile (i.e. female) sexual morph in most gynodioecious systems (Bailey and Delph 2007a). Thus, differences in cytoplasmic factors between females and hermaphrodites may lead to differences in seed fitness, as suggested in other studies of gynodioecious plants (Jolls and Chenier 1989, Ashman 1992). However, Delph and Mutikainen (2003) found that seedling survival in the gynodioecious *Silene acaulis* differed between maternal sexes even when maternal parents shared the same cytotype, indicating that factors beyond cytoplasmic inheritance likely contribute to seed fitness differences. Because the seed coat is formed from ovule tissue, its genetic composition is the same as the maternal plant (Baskin and Baskin 2014, Radchuk and Borisjuk 2014). Thus, the interaction we found between maternal plant sex and scarification may reflect differences in maternal seed coat properties, though further research is needed to determine whether morphological differences occur or how they might affect dormancy in *S. campestris*.

Seed dormancy and germination performance may also be influenced by cytonuclear interactions (Boussardon et al. 2019). Although the negative pleiotropic effects of *Rf* alleles (i.e. the cost of male fertility restoration) are expected to primarily affect pollen fitness (Bailey 2002, Delph et al. 2007), a cost to seed fitness is reported in some gynodioecious species (de Haan et al. 1997, Del Castillo and Trujillo 2009). Delph and Mutikainen (2003) suggest that in *Silene acaulis*, differences in the survival of seeds produced by females and hermaphrodites may result from such pleiotropic effects of *Rf* alleles. In maize (*Zea mays*), a monoecious species, several nuclear restorers of S-type CMS are reported to have deleterious effects on kernel size and development (Gabay-Laughnan et al. 1995). Despite its theorized importance to the maintenance of gynodioecy (Bailey and Delph 2007a, 2007b), few studies have assessed the cost of male fertility restoration in gynodioecious systems (Bailey 2002, Dufaÿ et al. 2008, Del Castillo and Trujillo 2009, Case and Caruso 2010, Caruso and Case 2013). Further research is needed to assess potential interactions between *Rf* alleles and seed dormancy characteristics, such as seed coat durability and physiological dormancy mechanisms.

Beyond genetically inherited factors, maternal environment and resource allocation may also shape seed fitness (Baskin and Baskin 1973). Chemicals produced by the maternal plant can affect offspring characteristics such as seed dormancy (Battle and Whittington 1971, Baskin and Baskin 2014). Sex-specific responses to environmental conditions are widely reported in sexually dimorphic plants (Hultine et al. 2016, Varga and Soulsbury 2020). If divergent sexual functions are associated with sex-specific resource demands, then sexes may differ in their acquisition and allocation of such resources (Delph 1999, Obeso 2002, Hultine et al. 2016, He et al. 2025). For example, the availability of environmental resources can shape the extent to which hermaphrodites invest in female function (Delph 2003, Spigler and Ashman 2011, Vaughton and Ramsey 2012, Cuevas et al. 2014). The resource environment of the maternal plant can influence the dormancy signals passed on to seeds (Baskin and Baskin 2014), and in gynodioecious plants, sexual dimorphism in resource acquisition and allocation may alter the maternal environment experienced by developing seeds (Ashman 1992, 1994).

### Evolutionary implication

Similar seed viability and mass for both sexes suggests that female advantage, and thus the maintenance of gynodioecy in *S. campestris*, is not explained by such differences in seed quality. However, the adaptive significance and evolutionary implications of differential dormancy patterns between maternal sexes remain speculative. For example, if there is a fitness cost associated with early germination, then stronger dormancy in seeds produced by females may contribute to female advantage. Alternatively, variation in dormancy strength among hermaphrodite-produced seeds may represent a bet-hedging strategy, reducing the risk that all offspring encounter unfavourable growing conditions (de Jong and Klinkhamer 2005, Kortessis and Chesson 2019, Postma and Ågren 2022), or it may arise as a fitness cost of the negative pleiotropic effects of *Rf* alleles, as noted above. Finally, because dormancy can have cascading effects across life-history traits, influencing the timing of germination, growth, and flowering (Postma and Ågren 2022), sex-specific differences in tradeoffs among these components may result in differing selective pressures on dormancy.

Differences between females and hermaphrodites in seed yield probability, year-to-year seed yield variation, and/or the survival rates of seeds in the soil may give rise to differing optimal seed dormancy strategies (Venable 1989). A variety of genetic and ecological factors may contribute to such differences. For example, females are generally expected to experience greater pollen limitation (Maurice and Fleming 1995, Dufay and Billard 2012) and to exhibit less plasticity than hermaphrodites in their allocation of resources to seed production (Delph 2003, Dorken and Mitchard 2008). Further work is needed to determine how such sex-specific differences in reproductive success influence the evolutionary outcomes of seed dormancy, which is shaped by interactions between variability in seed yield and germination (Kortessis and Chesson 2019).

## Conclusion

In this study, we evaluated how maternal sex affects seed characteristics in *S. campestris*, comparing dormancy, mass, and viability of seeds from female and hermaphrodite plants. Overall, germination was highest when seeds received a combination of scarification and 12-week cold stratification, suggesting *S. campestris* germination may be influenced by physical and physiological dormancy. While scarification and stratification promoted germination in seeds from both sexes, we found that seeds from females and hermaphrodites differed in their germination responses to dormancy-breaking treatments. Specifically, seeds produced by hermaphrodites were more easily released from dormancy than seeds produced by females. These differences do not appear to be driven by seed quality, as seed viability and mass were similar between maternal sexes. Our results highlight the need for further research to clarify the ecological and evolutionary implications of sex-specific maternal effects on seed dormancy and germination in gynodioecious systems.

## Supplementary Material

plag031_Supplementary_Data

## Data Availability

Raw data and R code are available online at https://osf.io/ha5uw/overview?view_only=31de0a2c5c75407c9d76b5ca8da919b2.
