# Supplementary material for: Maternal sex influences seed dormancy in a gynodioecious species
Source: AoB Plants. 2026 Jul 3;18(4):plag031. doi: 10.1093/aobpla/plag031 (PMC13520120; doi:10.1093/aobpla/plag031)
Supplement: Supplementary file 1 [file plag031_supplementary_data.docx]

| **Population site name** | **Latitude** | **Longitude** | **Populations size** | **Female frequency (%)** | **Harvest date (MM/DD/YYYY)** |
| --- | --- | --- | --- | --- | --- |
| Glaser Drive | 44.51921 | -123.028 | 108 | 34.26 | 06/10/2024 |
| Canemah Bluff Nature Park | 45.3449 | -122.626 | 81 | 66.67 | 07/15/2024 |
| Isik Creek | 45.35456 | -123.192 | 30 | 66.67 | 07/19/2024 |

**Table S1.** Characteristics of the 3 *Sidalcea campestris* populations used to source seeds used in this study, including population name, latitude, longitude, size, female frequency, and harvest date.
